# US-wide equine strongylid egg count data demonstrate seasonal and regional trends

**DOI:** 10.1017/S0031182024000489

**Published:** 2024-05

**Authors:** Martin K. Nielsen, Paul Slusarewicz, Tetiana A. Kuzmina, Matthew J. Denwood

**Affiliations:** 1 M.H. Gluck Equine Research Center, Department of Veterinary Science, University of Kentucky, Lexington, Kentucky, USA; 2 Parasight System, Inc, 1532 North Limestone Road, Lexington, Kentucky, USA; 3 I.I. Schmalhausen Institute of Zoology NAS of Ukraine, Bogdan Khmelnytsky Street 15, Kyiv, Ukraine; 4 Institute of Parasitology, Slovak Academy of Science, Hlinkova 3, Košice 04001, Slovak Republic; 5 Department of Veterinary and Animal Sciences, University of Copenhagen, Denmark

**Keywords:** horse, monitoring, nematode, parasite, sample

## Abstract

Equine strongylid parasites are ubiquitous around the world and are main targets of parasite control programmes. In recent years, automated fecal egg counting systems based on image analysis have become available allowing for collection and analysis of large-scale egg count data. This study aimed to evaluate equine strongylid fecal egg count (FEC) data generated with an automated system over three years in the US with specific attention to seasonal and regional trends in egg count magnitude and sampling activity. Five US regions were defined; North East, South East, North Central, South Central and West. The data set included state, region and zip code for each FEC. The number of FECs falling in each of the following categories were recorded: (1) 0 eggs per gram (EPG), (2) 1 ⩽ 200 EPG, (3) 201 ⩽ 500 EPG and (4) >500 EPG. The data included 58 329 FECs. A fixed effects model was constructed fitting the number of samples analysed per month, year and region, and a mixed effects model was constructed to fit the number of FECs falling in each of the 4 egg count categories defined above. The overall proportion of horses responsible for 80% of the total FEC output was 18.1%, and this was consistent across years, months and all regions except West, where the proportion was closer to 12%. Statistical analyses showed significant seasonal trends and regional differences of sampling frequency and FEC category. The data demonstrated that veterinarians tended to follow a biphasic pattern when monitoring strongylid FECs in horses, regardless of location.

## Graphical abstract

**Figure:**
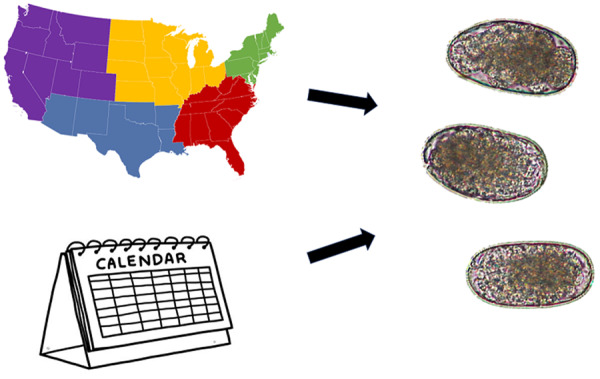

## Key findings

- Data consisted of 58 329 equine strongylid fecal egg counts determined during 2019–2022.
- Veterinarians consistently performed more fecal egg counts in the spring and autumn.
- Egg counts tended to be higher during the spring and autumn.
- Mean egg counts were generally lower in the West region.
- Overall, 18% of horses contributed 80% of the total strongylid egg output across the study.

## Introduction

Equine strongylid parasites are known to infect grazing horses across the world. While infections are most often asymptomatic (Nielsen *et al*., 2021), cyathostomins possess substantial pathogenic potential as the cause of Larval Cyathostominosis, a disease complex that in its acute form is associated with a guarded to poor prognosis for survival (Love *et al*., 1999; Peregrine *et al*., 2006; Lawson *et al*., 2023). Traditional approaches for control of these parasites have been based on frequent anthelmintic treatments administered to all horses present on a given farm and applied at fixed intervals year-round (USDA, 1999; Lloyd *et al*., 2000; O'Meara and Mulcahy, 2002). However, due to development of anthelmintic resistance to all available drug classes in equine nematode parasites (Nielsen, 2022), it is now recommended this approach be abandoned and instead replaced by surveillance-based strategies for equine parasite control (Kaplan and Nielsen, 2010; ESCCAP, 2019; Nielsen *et al*., 2019; Rendle *et al*., 2019) that make routine use of parasite fecal egg counts (FECs) within parasite control programmess. To facilitate this, an automated image analysis-based platform has been developed for determining equine strongylid fecal egg counts (Slusarewicz *et al*., 2016; Cain *et al*., 2020). This system is now commercially available, units are placed in veterinary practices across the USA, and all egg count data are centrally stored in a company cloud database. This facilitates the collection of large datasets in a standardized manner using the same technology in different locations, which has substantial potential for improving surveillance-based strategies for parasite control.

The recommendation to monitor strongylid egg shedding status in horses is based on the observations that strongylid FECs are highly over-dispersed among horses, following the Pareto principle or the law of the vital few, which describes a wide range of phenomena where a large proportion of the total outcome is represented by a small proportion of individuals. Indeed, it has been widely reported that around 20% of mature horses shed 80% of the total egg output from the population (Relf *et al*., 2013; Wood *et al*., 2013; Lester *et al*., 2018; Nielsen *et al*., 2018), and that this pattern is consistent in individual horses across time (Nielsen *et al*., 2006, Becher *et al*., 2010; Wood *et al*., 2013; Scheuerle *et al*., 2016). Consequently, treating the high-shedding subset of the population can effectively lower the overall parasite egg output (Kaplan and Nielsen, 2010) and, thus, decrease pasture contamination effectively without having to treat the entire population. A previous study conducted in the USA confirmed a strongylid 80/20 shedding pattern, but also suggested regional differences, with horses present in Western states (Arizona, California, Colorado, Montana, Oregon and Wyoming) displaying a higher degree of over-dispersion than horses in other states (Nielsen *et al*., 2018). It has been speculated that climatic differences may be responsible for these observations, although data supporting this are relatively sparse (Nielsen *et al*., 2018).

Equine strongylid egg shedding has been demonstrated to fluctuate by season in some studies (Poynter, 1954; Duncan, 1974; Wood *et al*., 2013; Nielsen *et al*., 2021), but not in others (Lester *et al*., 2018; Steuer *et al*., 2022). However, regardless of whether these fluctuations occur or not, the general recommendation is to focus egg count monitoring and anthelmintic treatments around the active parasite transmission season (ESCCAP, 2019; Nielsen *et al*., 2019) so that treatments are aimed at reducing pasture contamination. Cyathostomin modeling studies have demonstrated how parasite infection dynamics are largely driven by climate and seasonality (Leathwick *et al*., 2015, 2019), and parasite transmission patterns have been predicted to differ substantially within the USA (Leathwick *et al*., 2019). Thus, it appears that the optimum timing for fecal sampling and associated anthelmintic treatment, if needed, may differ between USA regions. However, no national-level information is currently available regarding the time of year that USA veterinarians typically determine equine strongylid FECs, or whether there are seasonality patterns in sample positivity or egg count magnitude.

This study aimed to evaluate equine strongylid FEC data generated with an automated system over three years in the USA with specific attention to seasonal and regional trends in egg count magnitude and sampling activity. Furthermore, the study also aimed to describe overdispersion patterns across years, seasons and geographic locations.

## Materials and methods

### Data source

Strongylid fecal egg count data were obtained from an automated image analysis based fecal egg counting system (Parasight System, Lexington, KY, USA). Counts were determined by 140 units placed in a subset of veterinary practices agreeing to contribute data for this study. The practices were located in 37 US states, and the data were collected during the calendar years of 2019–2022. Veterinary practices were anonymized for the analyses, and sample dates were provided at a resolution of calendar month and year, preventing the identification of individual farms. The sample data were provided as transformed counts in eggs per gram (EPG) of feces, and due to the complex software procedure used by the units to determine the volume of sample to examine (as well as the certainty threshold cutoff for differentiating eggs from detritus), it was not possible to back-transform the data into statistically valid numbers of eggs counted prior to conversion. However, for ease we refer to these transformed counts simply as fecal egg counts (FEC) in this article. In addition to the FECs and month/year, the following information was provided at sample level: zip code of the veterinary practice, anonymous ID of the camera (a proxy for veterinary practice) and horse age category (above or below 1 year of age). The egg counting app does not systematically collect information on anthelmintic treatment and/or treatment efficacy.

### Data handling

The data were first cleaned to remove observations associated with test/demonstration machines, based on identifiers associated with these units. A pseudo-date corresponding to the 15th day of the provided month/year was generated to facilitate subsequent analyses, and the following US regions were defined based on the provided state: North East (Maine, New Hampshire, Vermont, Massachusetts, Connecticut, New York, Pennsylvania, Delaware and Maryland), South East (Kentucky, West Virginia, Virginia, North Carolina, Tennessee, South Carolina, Georgia, Alabama, Mississippi and Florida), North Central (North Dakota, South Dakota, Nebraska, Kansas, Minnesota, Iowa, Missouri, Wisconsin, Illinois, Michigan, Indiana and Ohio), South Central (Arizona, New Mexico, Texas, Oklahoma, Arkansas and Louisiana) and West (Washington, Oregon, California, Idaho, Nevada, Montana, Wyoming and Colorado).

The sample data were then aggregated at the level of veterinary practice and pseudo-date to ascertain the following:

- Mean FEC
- Total number of FECs
- Number of FECs equal to 0 (zero FEC)
- Number of FECs >0 and ⩽200 (low FEC)
- Number of FECs >200 and ⩽500 (moderate FEC)
- Number of FECs >500 (high FEC)

The variables of the US state and regional location of each veterinary practice were also included as additional columns. We refer to this dataset as the aggregated data throughout the remainder of the article. The aggregated data were then summarized by calendar month/year to obtain the number of veterinary practices conducting one or more FECs during that month, as well as by veterinary practice to determine the number of separate calendar months/years during which each veterinary practice generated one or more FEC. This was done to identify the general consistency of sampling intensity over the time period, as well as to identify the veterinary practices that determined FECs most consistently throughout the year. We defined the restricted time period as the 24 months between April 2020 and March 2022, and the restricted veterinary practices as those that contributed data for at least 18 (75%) of these months.

### Descriptive analyses

Summary plots were created for the observed strongylid FEC, stratified by US region (as defined above). These included empirical cumulative distribution function (ECDF) plots of the raw data, stacked bar charts showing the number of zero, low, moderate and high FECs by calendar month and a temporal plot of the mean FEC. The proportion of animals responsible for 80% of the total strongylid egg count was calculated to evaluate the ‘80/20 rule’ of strongylid egg shedding. This was done by sorting the observed counts in descending order, calculating a running cumulative total, and then converting this to a cumulative proportion of the total by dividing by the sum of the counts (so that the highest count had a cumulative proportion of 0, and the lowest count had a cumulative proportion of 1). The observation number corresponding to the closest cumulative proportion to 0.8 was extracted, before dividing by the number of observations to obtain the proportion of animals that had contributed 80% of the total FEC. This process was repeated on different subsets of the data, including stratifying by US region and year.

All descriptive analyses were performed using tidyverse packages (Wickham *et al*., 2019) in R version 4.3.0 (R Core Team, 2023).

### Statistical models

Two sets of statistical models were used to estimate seasonal trends based on the aggregated data from the restricted time period. For Model 1, we fitted the observed total number of FECs in each calendar month/year and US region using a fixed effects model with a negative binomial response. For Model 2, we fitted the number of FECs that were below/above a given threshold value based on the aggregated data from the restricted time period and restricted veterinary practices to a mixed effects logistic regression model. Three different thresholds were used for separate model fits as follows: zero *vs* low/moderate/high FEC (Model 2A), zero/low *vs* moderate/high FEC (Model 2B) and zero/low/moderate *vs* high FEC (Model 2C). The three mixed effects models used random effects of veterinary practice and month/year/veterinary practice to facilitate clustering in the data (we expected that some of these clusters would contain data from the same farm, but this information was not available in the data we obtained).

All 4 models included fixed effects of US region, as well as 2 sine waves: one with an annual period and one with a biannual (6-month) period. These sine waves were intended to decompose the seasonal trends in the data so that 4 parameters representing the timing of annual and biannual peaks and the amplitude of the 2 seasonality effects could be estimated. Additional fixed effects representing the US state and interaction between US state and the 4 seasonality parameters were also included in all models. For Model 1, we also included a linear trend term and interaction with US state to capture changes in uptake of the automated egg counting system over time.

All statistical models were fitted using R version 4.3.0 (R Core Team, 2023), with fixed effects models implemented using the MASS package (Venables and Ripley, 2002), mixed effects logistic regression models using the lme4 package (Bates *et al*., 2015) and ANOVA tables using the car package (Fox and Weisberg, 2019).

## Results

### Descriptive statistics

The data contained 58 329 FEC observations from 141 different veterinary practices in 37 US states between July 2019 and March 2022. These were distributed by region as follows: North East: 44 units, South East: 42, North Central: 22 units, South Central: 12 units and West: 20 units. The image analysis method has a non-integral variable multiplication factor, such that FECs of e.g. 5, 10, 12, 15, 20 and 25 were approximately 20 times more frequently observed than FECs of e.g. 4, 9, 13, 18 and 22, and FECs of e.g. 1, 2, 3, 6, 8, 11, 16 and 17 were not observed. The median number of FECs per veterinary practice was 198 (range 1–3068). Each veterinary practice contributed data from median of 17 out of the potential maximum of 33 calendar months (range 1–32). Excluding the data from before April 2020 (with relatively few recordings) resulted in 54 137 FEC observations from 140 different veterinary practices within the restricted time period, each contributing a median of 17.5 out of the potential maximum of 24 calendar months (range 1–24). A total of 70 (50%) veterinary practices contributed data from at least 18 of these 24 calendar months, including 29 (21%) contributing data in all months.

**Figure 1.**
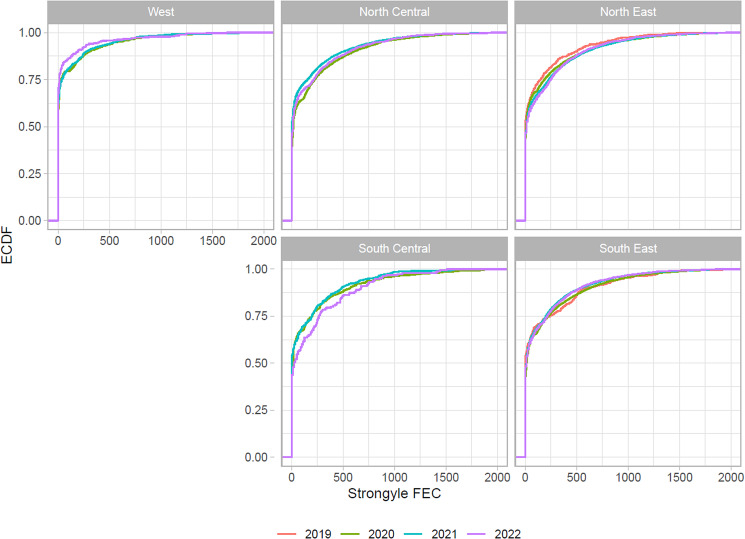
Empirical cumulative distribution function (ECDF) plots of the observed strongyle fecal egg count data, stratified by region and year. Note that the x axis is truncated to a maximum of 2000 eggs per gram (EPG).

Empirical cumulative distribution function (ECDF) plots for the observed strongylid FECs stratified by region and year, are shown in Fig. 1. Approximately 50% of the observed FECs were zero in each of the regions/years, except for West, where this proportion was somewhat higher (around 60%). The vast majority of counts was below 2000 EPG, but occasional higher counts were observed (up to 5576, 4833, 4030, 5840 and 3217 in North Central, Northeast, South Central, Southeast and West, respectively).

**Figure 2.**
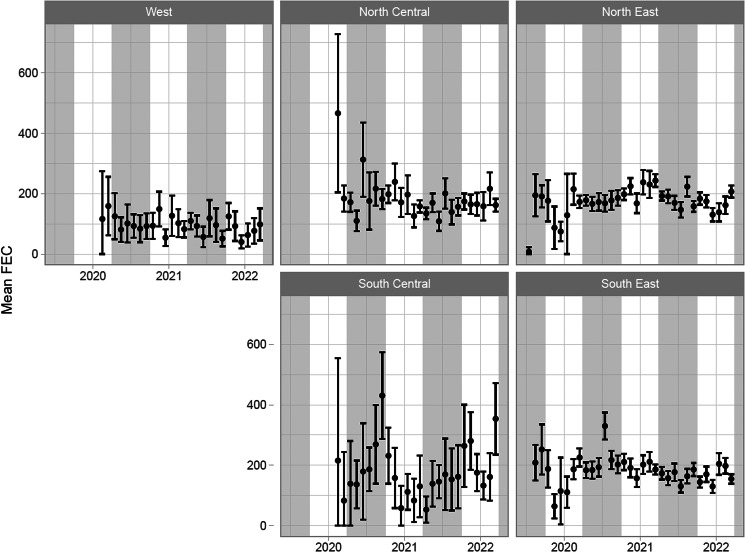
Overall mean fecal egg count (FEC) by US region over the time period covered by the data. Error bars represent crude 95% confidence interval estimates for the mean (lower confidence intervals truncated to zero where necessary). Year markings indicate the 1st January, and shaded background areas indicate the summer season (April–September, inclusive).

The observed overall mean FEC by calendar month/year and overall frequency of zero/low/moderate/high FECs are shown in Fig. 2 and Supplementary Fig. 1, respectively. Relatively few observations were available from before April 2020, and markedly fewer observations were available from South Central and West compared to the other regions. Mean FEC was generally lower for West compared to the other regions.

**Figure 3.**
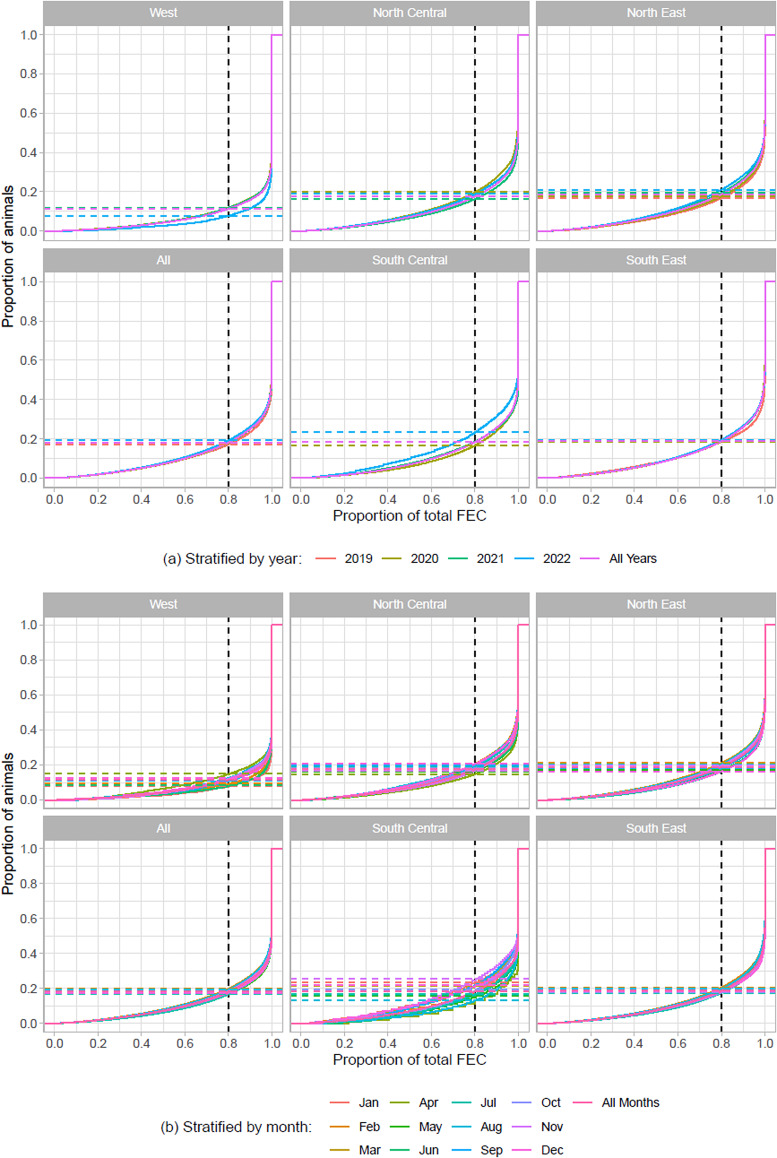
Illustration of the proportion of horses shedding 80% of the overall strongylid egg output (the ‘80/20 rule’), stratified by US region and year (a) / month (b).

Analysis of the ‘80/20’ strongylid FEC distribution rule for these data showed that the overall proportion of animals responsible for 80% of the total FEC output was 18.1% (Fig. 3). This figure was remarkably consistent over years and months, and all regions except for West, where the proportion was closer to 12% (Fig. 3). Closer analysis of the West region revealed that there were 4 practices with > = 200 samples, of which one was quite typical of the 18/80 distribution, but the other 3 were not (Supplementary Fig. 2).

### Sampling frequency

**Figure 4.**
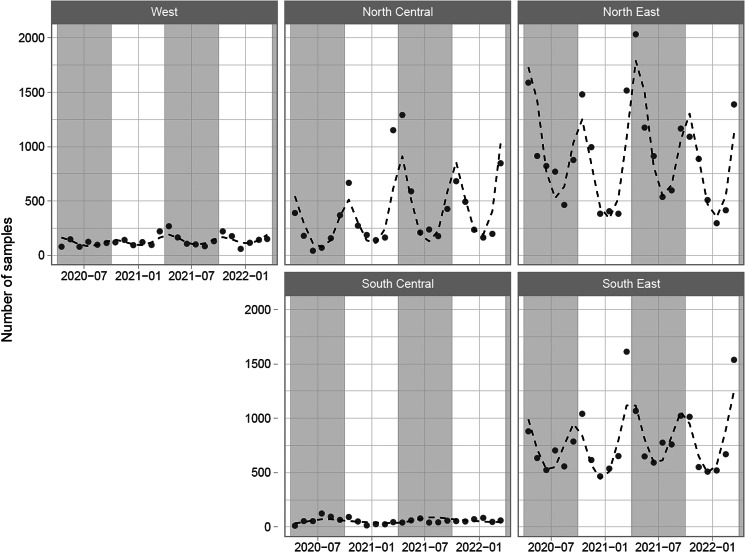
Fitted predictions of the mean number of samples over time (Model 1; dashed lines) overlaid with observed data (points) for all veterinary practices over the restricted time range, showing seasonal patterns and temporal trends in the number of samples taken within each US region. Year-month markings indicate the 1st of that month, and shaded background areas indicate the summer season (April–September, inclusive).

Model 1 showed significant seasonal trends as estimated using ANOVA tests for both annual and biannual sine waves. There was also a significant effect of US region and a significant log-linear trend, as well as significant interactions between US region and annual/biannual/linear temporal effects. However, the timing of peaks was qualitatively similar between regions (Fig. 4). Full details of the model results are given in Supplementary File 1.

### Proportions of egg count categories

**Figure 5.**
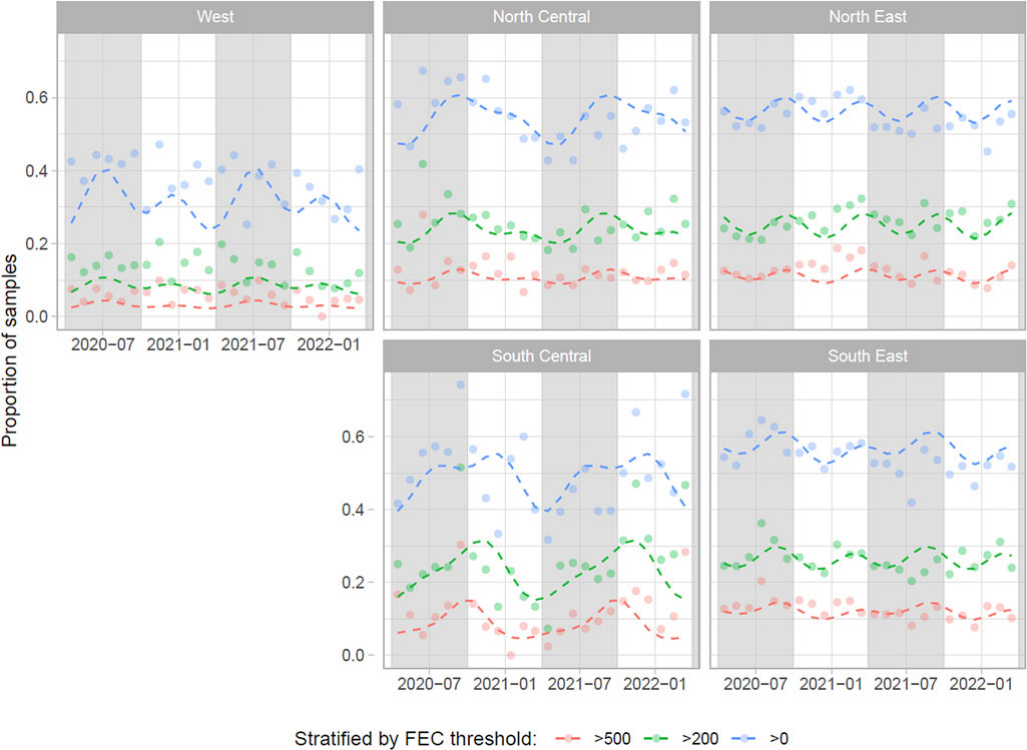
Fitted predictions of the proportion of fecal egg counts (FECs) over the specified threshold (Model 2a-c; dashed lines) overlaid with observed data (points) for restricted veterinary practices over the restricted time range, showing seasonal patterns in FEC classifications within each US region. Year-month markings indicate the 1st of that month, and shaded background areas indicate the summer season (April–September, inclusive).

Models 2a-c all showed significant seasonal trends for both annual and biannual sine waves (as determined by ANOVA tests), as well as US region and the interaction between these. Random effect standard deviation estimates were between 0.44 and 0.54 for all models for both veterinary practice and month/year/veterinary practice (the observation-level random effect). Overall seasonal trends were similar for different thresholds within the same region but differed between regions (Fig. 5). In general, a larger proportion of samples had counts exceeding 0, 200 and 500 EPG during the spring and autumn months across regions. Full details of the model results are given in Supplementary File 2.

## Discussion

This study is the first to investigate equine strongylid FEC patterns across the USA within and between years. The data confirmed a consistent ‘80/20 rule’ for strongylid egg count distributions across a very large dataset and demonstrated seasonal patterns of FEC magnitude. Furthermore, the study demonstrated that US veterinarians in most regions have a strong tendency to follow a biphasic pattern when monitoring for strongylid fecal egg shedding in their clientś horses with a majority of samples processed during spring and autumn months.

It was remarkable that the data from the West region were substantially different from the other 4 regions. The West region data had a higher proportion of zeros (Supplementary Fig. 1) and demonstrated a larger degree of overdispersal with 12% of the horses shedding 80% of the eggs (Fig. 3). It should be mentioned that due to the automated nature of the FEC data generated herein, such observed differences are less likely to be due to protocol differences and analyst experience than with traditional manual FECs. Overall, these observations agreed with a previous manual FEC study, which also found lower positivity and more pronounced overdispersal in this region (Nielsen *et al*., 2018). Possible reasons for these observations include climatic characteristics for this region, which tends to be more arid and often offers limited pasture access. This is expected to have a substantial impact on strongylid transmission dynamics and result in a much lower infection pressure compared to other regions. However, it should be acknowledged that the data from this region were sparse and only represented by a few practices, so results should be interpreted with caution. The data from the other 4 regions followed similar distributions with roughly about 18% of horses shedding 80% of the eggs (Fig. 3). Adding to this, we also demonstrated for the first time that these patterns were consistent between sampling months and years (Fig. 3). Taken together, these data confirm that a minority of horses contribute the primary proportion of pasture contamination, which supports surveillance-based parasite control strategies aimed at identifying high strongylid shedders and treating them accordingly.

The sampling frequency data demonstrated clear biphasic patterns across the studied regions, where most samples were analysed during spring and autumn months (Fig. 4). This is surprising given that climatic conditions are vastly different between regions, and conditions favouring strongylid transmission vary substantially (Leathwick *et al*., 2019). As a result, strongylid transmission seasonality likely varies largely between regions, which means that the optimal timing for treatments aimed at reducing pasture contamination should vary as well. However, the data from this study suggest that most veterinarians appeared to follow a one-size-fits-all calendar-based approach. Depending on the drug class chosen and its anthelmintic resistance profile in the given strongylid population, an anthelmintic can be expected to suppress egg output for a limited number of weeks, if at all (Nielsen, 2022). If treatments are administered outside what can be considered the strongylid parasite transmission season, they may have little or no effect on the infection pressure, since egg counts are likely to return to pretreatment levels before the season picks up again. It should be acknowledged, however, that information about the timing of possible anthelmintic treatment was not available within these data and fecal sampling may not always correspond to treatments being administered. Nonetheless, these results indicate a need for educating veterinarians in identifying the optimal timing for recommending fecal sampling and treatments aimed at suppressing strongylid egg output and subsequent pasture contamination.

As outlined in the introduction, some past studies have described clear seasonal patterns in strongylid egg shedding, while others have not. Given this, it was interesting to observe apparent differences in the proportion of FECs exceeding various thresholds in this study (Fig. 2). While limited seasonal fluctuations were observed in the West and South Central regions, the other regions displayed more pronounced patterns (Fig. 4). It could be hypothesized that these observations may in part reflect climatic influences on parasite transmission, but it should, again, be acknowledged that information about anthelmintic treatments administered was not available in this study. As anthelmintic treatment regimens are expected to have a significant impact on FEC results obtained, no firm conclusions can be drawn at this stage. Similarly, we have no information about age distributions of horses tested by region, which could also affect study outcomes. Furthermore, strongylid species composition could be hypothesized to contribute to these patterns but were not accounted for in this study. Nonetheless, the study found apparent differences in positivity patterns between regions, which should be investigated further in future studies.

In addition to the study limitations mentioned in previous sections, it should be mentioned that the identity of individual horses was anonymized, which meant that it was not possible to discern if FECs from some horses appeared multiple times in the dataset. Furthermore, there was no information about management, breed, use or sex of the horses, which limited opportunities for comprehensive multivariate statistical analyses. In addition, the variable multiplication factor of the automated egg counting method excluded the possibility of fitting count-based parametric models to the data, and linear models based on transformations of the data showed extremely poor fit and were therefore not included here. However, the sampling frequency data are not affected by these limitations because they are merely a measure of the number of samples processed across time. Considering this, the sampling frequency data should be considered the strongest component of this dataset, as they describe sampling behaviour of equine veterinarians across the USA.

In summary, this study made use of a large dataset and is the first to demonstrate a consistent pattern of 80/20 distribution of strongylid FECs across different US regions and over the course of 3 years. Furthermore, the data demonstrated that veterinarians tend to concentrate their parasite surveillance efforts during spring and autumn months regardless of location and climate. Finally, the data displayed some seasonality with regards to sample positivity and FEC magnitude level. More studies are needed to better understand factors affecting equine strongylid egg shedding patterns.

## Supporting information

Nielsen et al. supplementary material 1 Nielsen et al. supplementary material

Nielsen et al. supplementary material 2 Nielsen et al. supplementary material

Nielsen et al. supplementary material 3 Nielsen et al. supplementary material

Nielsen et al. supplementary material 4 Nielsen et al. supplementary material

## Data Availability

The full data set can be made available upon request to the corresponding author.

## Supplementary material

The supplementary material for this article can be found at https://doi.org/10.1017/S0031182024000489.
