# Supplementary figures and images for: US-wide equine strongylid egg count data demonstrate seasonal and regional trends
Source: Parasitology. 2024 Apr 17;151(6):579–86. doi: 10.1017/S0031182024000489 (PMC11428020; doi:10.1017/S0031182024000489)

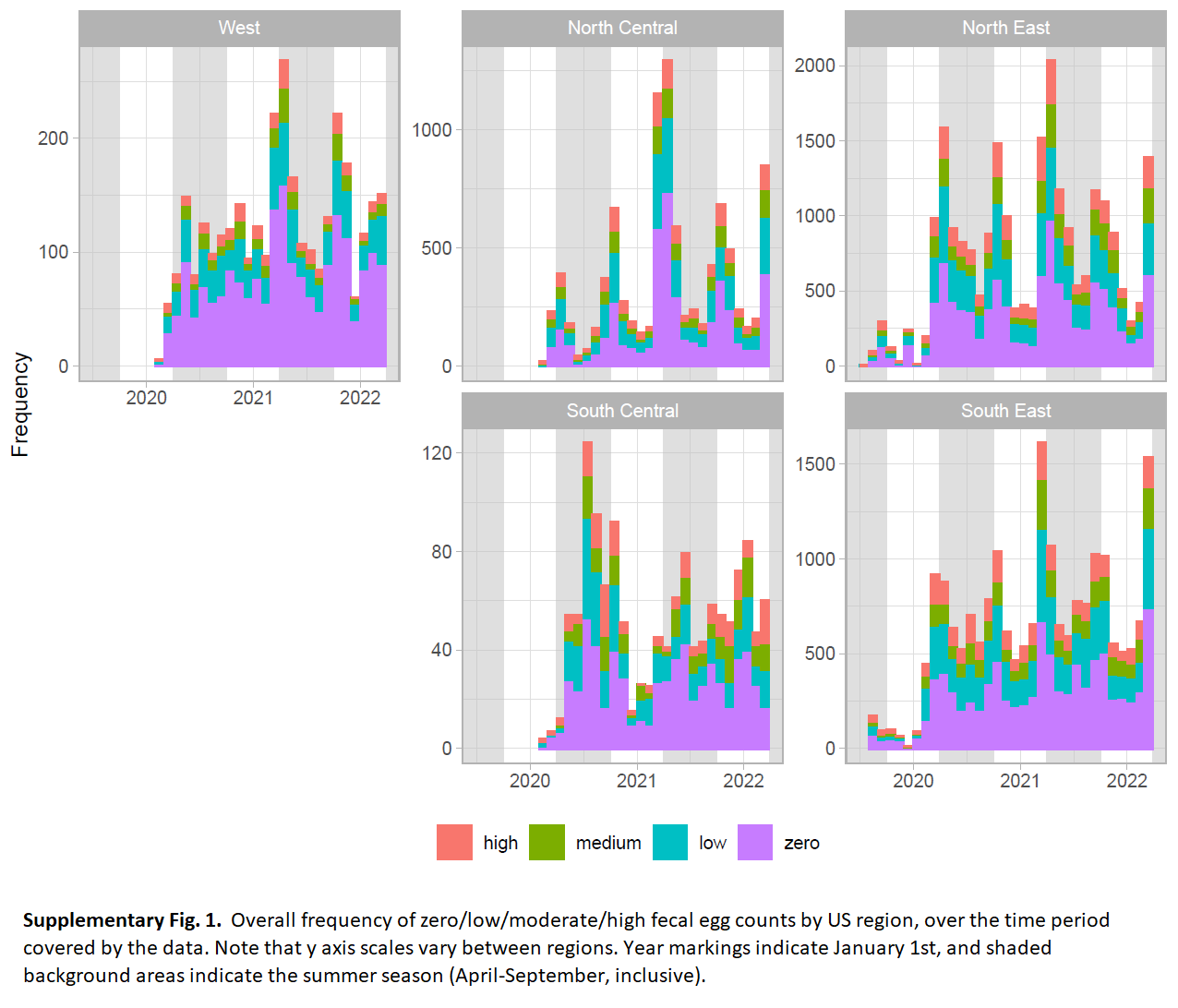

Supplement: Nielsen et al. supplementary material 1 — Nielsen et al. supplementary material [file S0031182024000489sup001.tif]

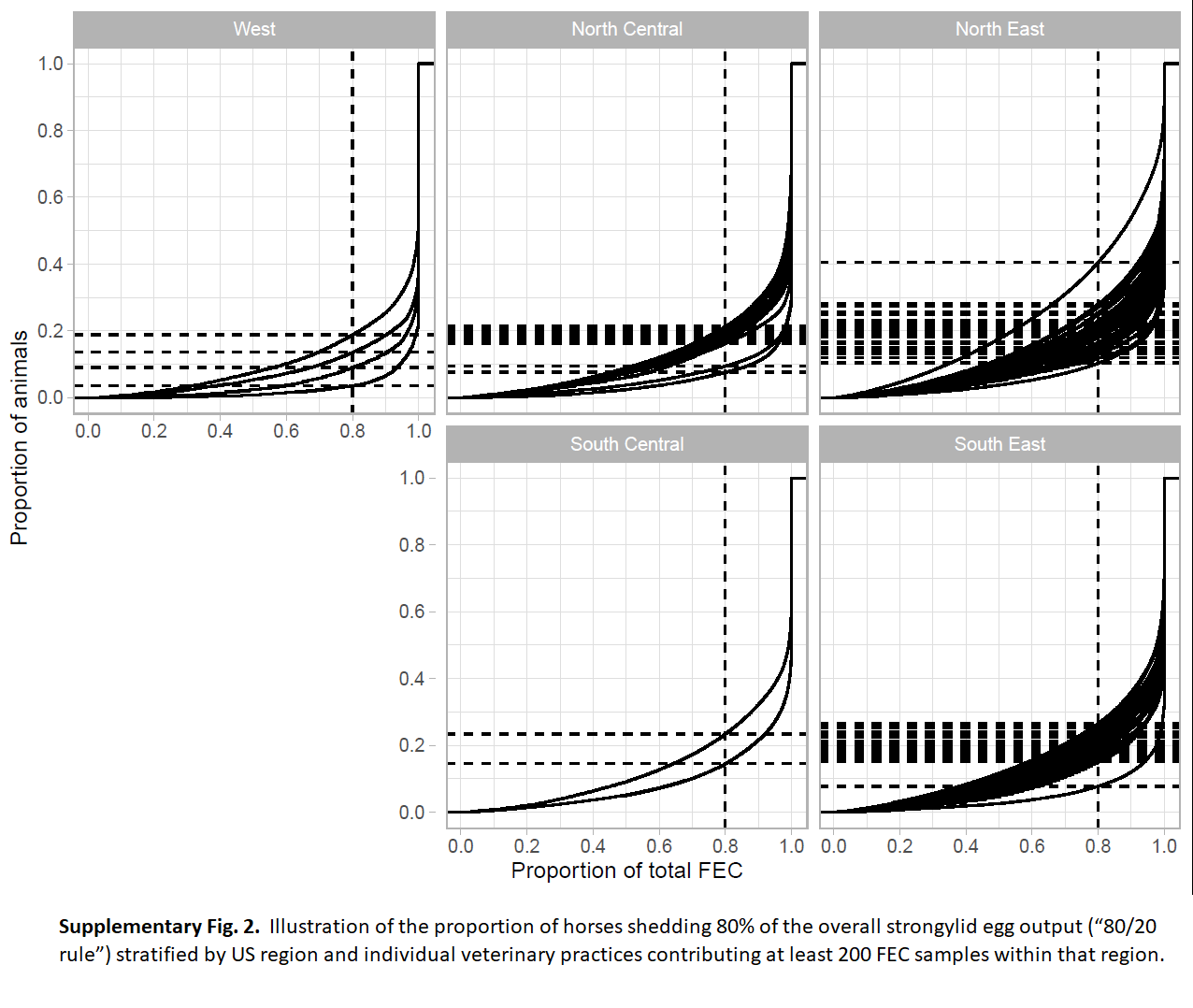

Supplement: Nielsen et al. supplementary material 2 — Nielsen et al. supplementary material [file S0031182024000489sup002.tif]
